# A new sensitive UPLC-MS/MS method for the determination of cucurbitacin B in rat plasma: application to an absolute bioavailability study

**DOI:** 10.1039/c8ra05941a

**Published:** 2018-09-05

**Authors:** Ya Xiao, Qiang Zhao, Qian Wu, Jinhua Chang, Hefei Xue, Cuizhe Liu, Xigang Liu

**Affiliations:** Hebei Key Laboratory of Research and Development for Chinese Medicine, Chengde Medical University Anyuan Road, Shuangqiao Chengde Hebei 067000 P. R. China liuxgmail@sina.com liucuizhexy@163.com

## Abstract

Cucurbitacin B (CuB) is a highly oxygenated tetracyclic triterpene, and a Biopharmaceutics Classification System (BCS) class IV drug used for the treatment of persistent hepatitis, chronic hepatitis, and primary liver cancer. Nevertheless, CuB has low solubility and low permeability, and is present at low concentrations in the human body. The aim of this study was to develop a method for the determination of CuB in plasma using ultra-performance liquid chromatography-mass spectrometry (UPLC-MS/MS) with estrone as an internal standard (IS), as well as to examine the pharmacokinetics and absolute bioavailability of CuB in rats. Plasma samples were processed by liquid–liquid extraction with ethyl acetate. Separation was achieved on a BEH C18 column (2.1 × 50 mm, 1.7 μm) at 35 °C using an isocratic mobile phase system with 0.1% formic acid–acetonitrile (50 : 50, v/v) at a flow rate of 0.3 mL min^−1^. The detection was performed using a multiple reaction monitoring mode *via* a positive electrospray ionization interface. The calibration curves showed good linearity (*r* = 0.9998) within the tested concentration ranges. The lower limit of quantification for plasma was 0.05 ng mL^−1^; the matrix effect of CuB and IS was 94.19–99.42% and 100.83%, respectively. The mean extraction recoveries from plasma were 85.34–90.53%. The intra-day and inter-day accuracies and precision deviations were within ±15%, which was in line with the allowable range of accuracy. In addition, the stability of the method was also verified. The absolute bioavailability of orally administered CuB in rats was 1.37%. To sum up, the presented method was determined to be suitable for the quantitation of CuB in rat plasma. Also, the absolute bioavailability observed in the present study suggested that it was necessary to change the dosage form to improve bioavailability, or to improve this by other means.

## Abstract 2

A method was developed for the determination of CuB in plasma using UPLC-MS/MS with estrone as an internal standard, and the pharmacokinetics and absolute bioavailability of CuB in rats were examined.

## Graphical abstract

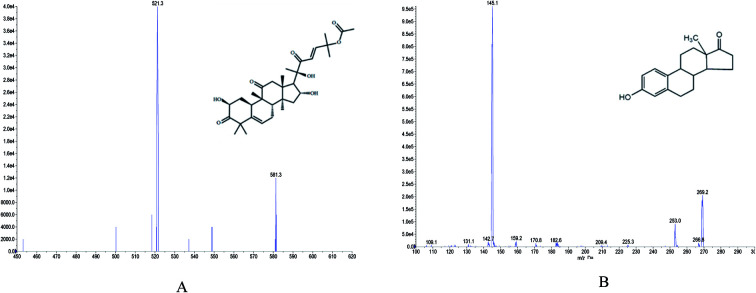

## 1. Introduction

Cucurbitacins are highly oxygenated, tetracyclic triterpenes that can be isolated from members of the Cucurbitaceae family such as cucumber, watermelon, and zucchini. More than 18 kinds of cucurbitacins can be found in nature, including cucurbitacin B.^1^ CuB is recognized as the major active ingredient which possesses a broad range of pharmacological properties such as hepatoprotection, anti-tumor effects and immune enhancement.^2,3^ Until now, CuB has been used for the treatment of persistent hepatitis, chronic hepatitis, and primary liver cancer, as it has significant anti-inflammatory activities and antitumor effects.^4^ The exact mechanism of the anti-tumor effect of CuB appears to be related to induction of cell apoptosis and cell cycle arrest, cleavage of cytoskeletons, and blockage of cell signaling pathways.^5–8^ Nevertheless, there are relatively few studies on the precise pharmacokinetic mechanism of CuB. So far, many chromatographic systems for the determination of CuB content have been developed.^9,10^ Among these, only a few can be used for the determination of plasma samples. For example, Dou *et al.* examined the content of CuB using HPLC.^11^ In addition, Wang *et al.* used an HPLC-UV method for the quantification of CuB with a lower limit of quantification (LLOQ) of 1.0 μg mL^−1^ in human serum.^12^ However, these approaches offered low sensitivity and poor specificity. Bajcsik *et al.* determined cucurbitacin B, E, I and E-glucoside by HPLC-MS with limits of quantification of 0.05–0.42 ng mL^−1^ and analysis was by Q1 scans with negative ionization.^13^ The emergence of new analytical methods such as UPLC-MS/MS and sample preparation advancements have increased sensitivity and efficiency.^14,15^ For example, UPLC-MS/MS has been recently used to determine the content and pharmacokinetics of CuB in rat plasma; the LLOQ of CuB in plasma measured by this method was 0.3 ng mL^−1^.^16^ Moreover, Wang *et al.* have adopted a combined UHPLC-MS/MS system to determine the content of CuB and CuE with limits of quantification of 1.60 ng mL^−1^.^17^ In previous experimental studies, a novel dual modality system *i.e.* UPLC-MS/MS was established for the quantitative analysis of CuB. Nonetheless, due to the complexity of condition optimization and the lower concentration of the sample in the body, determination of the CuB content and quality control in plasma after administration still need to be further investigated.

As a Biopharmaceutics Classification System (BCS) class four drug with low solubility and low permeability,^18,19^ CuB is present at low concentrations in the body of humans and animals. Therefore, it is very important to develop a more sensitive and simple way to determine its exact concentration in the body. Currently, there are no studies reporting the absolute bioavailability of cucurbitacin. Hence, the goal of this research was to establish and validate a highly selective and sensitive UPLC-MS/MS method for the determination of CuB in rat plasma, and to further explore its absolute bioavailability and its effect on plasma. Solid dispersion techniques were shown to be successful in improving the dissolution and bioavailability of poorly soluble drugs by reducing the particle size of the drug, by improving wettability and by forming amorphous particles. Accordingly, a solid dispersion of CuB should be further investigated.

## 2. Materials and methods

### 2.1. Chemicals and reagents

**Fig. 1:**
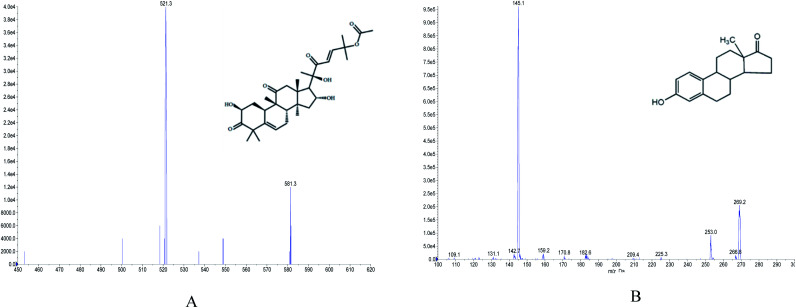
Chemical structures and mass spectra of CuB (A) and IS (B).

The chemical standard of CuB (purity > 96.9%, CAS111945-201301) was purchased from the National Institute for Food and Drug Control (Beijing, China); the internal standard (IS) estrone (purity > 98%, CAS C-021-170426) was supplied by Ruifensi Biotech Co. Ltd. (Chengdu, China). Their chemical constitutions are shown in Fig. 1. Acetonitrile and methanol were of MS grade and were purchased from the United States (Fisher Chemical). MS grade formic acid was procured from Spain (Fisher Scientific). Purified water for UPLC analysis was bought from Wahaha (Gaobeidian, Hebei, China).

### 2.2. Animals

Male adult Wistar rats were obtained from Beijing Vital River Laboratory Animal Technology Co. Ltd. All the animals were housed in an environment with a temperature of 22 ± 1 °C, a relative humidity of 50 ± 1% and a light/dark cycle of 12/12 h. Before starting the experiment, the rats randomly received standard chow and sterile water and were fasted for 12 hours before the start of the experiment.

### 2.3. UPLC-MS/MS conditions

A Waters Acquity UPLC Liquid Chromatograph included a Diode Array Detector, Sample Manager, Binary Solvent Manager and Empower 2 software. Chromatography separations were performed on an ACQUITY UPLC BEH C18 column (2.1 × 50 mm, 1.7 μm; Waters Corporation, Ireland), equipped with a guard column and eluted with an isocratic mobile phase system, which was composed of acetonitrile and 0.1% formic acid (50 : 50, v/v). The experimental conditions were as follows: the column temperature was kept at 35 °C, the flow rate was 0.3 mL min^−1^, the injection volume was 2 μL and the total running time was 2.5 min. The mass spectrometer was equipped with a Q-Trap® 5500 triple quadruple mass spectrometer (AB SCIEX, USA), which was operated in the positive multiple reaction monitoring (MRM) mode. The ESI source and MS/MS parameters were set as follows: ion spray voltage, +5500 V; curtain gas, 35 psi; temperature, 450 °C; ion source gas 1, 45 psi; ion source gas 2, 55 psi; the interface heater was on, and the collision gas was medium. The mass transitions were: CuB, *m/z* 581.3 → 521.3, IS, *m*/*z* 269.2 → 145.1, and the mass spectra of CuB and IS are shown in Fig. 1.

### 2.4. Preparation of working solutions, calibration standards and quality control (QC) samples

Stock solutions of the IS (106 μg mL^−1^) and CuB (120 μg mL^−1^) were formulated with methanol. Immediately before use, serial dilution of the stock solution with methanol provided the CuB working solutions. 848 ng mL^−1^ was obtained as a final concentration after diluting the IS stock solution. All the standard solutions were kept at 4 °C and were protected from light until further use.

The preparation of calibration standards for the specific steps: the appropriate amount of CuB working solution was transferred to 1.5 mL centrifuge tubes, dried, and consequently mixed with 50 μL aliquots of blank plasma to obtain the calibration concentrations. The resulting concentrations of CuB were 0.05, 0.1, 1.0, 25, 250, and 1000 ng mL^−1^. A double-blank plasma sample containing neither CuB nor the IS was prepared along with these calibration standards.

Quality control (QC) samples were prepared in three levels *i.e.* low (0.1 ng mL^−1^), medium (25 ng mL^−1^) and high (750 ng mL^−1^) concentration of CuB. The QC samples were prepared following the method for preparation of the calibrated sample.

### 2.5. Plasma sample treatment

All plasma samples were thawed at room temperature prior to analysis. The protein precipitation procedure was applied to the sample pretreatment. Briefly, the IS solution (50 μL, 848 ng mL^−1^) and 100 μL of methanol were mixed with 50 μL of the plasma sample in a 1.5 mL Eppendorf tube. The solution was then vortexed for 1.0 min, additionally mixed with 1.0 mL ethyl acetate, and consequently vortexed and centrifuged at 15 000 rpm for 3 min and 10 min, respectively. The supernatant fluid was then transferred to a new tube and was evaporated to dryness under a gentle nitrogen stream at 40 °C. The residue was redissolved with 100 μL of the mobile phase and centrifuged for 8 min. The amount of sample injected into the UPLC-MS system for analysis was 2 μL.

### 2.6. Method validation

The developed UPLC-MS/MS method was validated with respect to selectivity, linearity and LLOQ, matrix effects, recovery, accuracy, precision and stability according to the US Food and Drug Administration Bio-analytical Method Validation Guide.^20^

#### 2.6.1. Selectivity

Blank rat plasma from six different sources was compared with the corresponding spiked plasma. Specificity was evaluated by analyzing the potential interference of endogenous substances at the retention times of CuB and the IS. Each blank plasma sample was detected under the LC-MS system to ensure that the plasma was free from the interference of CuB and the IS.

#### 2.6.2. Linearity and LLOQ

The calibration samples were obtained by independently adding a series of IS solutions at different concentrations (50 μL, 848 ng mL^−1^), the working solution (50 μL), and 150 μL methanol to blank rat plasma (50 μL). Following the procedure described above, the final concentrations ranged from 0.05 to 1000 ng mL^−1^ of CuB. The calibration curves were constructed by linear regression of the area ratio for CuB/IS (*Y*-axis) and concentrations (*X*-axis) with a 1/*x* weighting factor. Concentrations of quality controls (QCs) and samples were calculated using the regression equation of the calibration curve. The LLOQ was determined from the lowest concentration (signal-to-noise ratio > 10) on the calibration curve with a precision of ±20% and an accuracy of ±20%.

#### 2.6.3. Matrix effects and recovery

The matrix effect was determined by comparing the peak areas of blank plasma from the spiked QC samples with those of the standard samples at equivalent concentrations (three different QC concentrations were used (*n* = 6, for each concentration)). The recovery was defined by comparing the peak areas of CuB extracted from plasma samples with the peak areas of the pure standard from which no extraction was performed. Each respective extraction recovery of CuB was measured at three different concentration levels (low, medium and high) of QC samples. The extracted samples were repeated six times at each concentration.

#### 2.6.4. The intra-, inter-day accuracy and precision

Accuracy was described as RE%, and it was determined by the degree of closeness between the measured concentration and the actual concentration of the label. Relative standard deviation (RSD%) was used to describe the precision of the determination. The QC samples were measured on the same day and after three days to calculate intraday precision and interday precision. The LLOQ was evaluated on the same day and for three consecutive validation days, with six replicates of low, medium and high concentrations of QC samples being evaluated. For the QC samples, the accuracy did not exceed 15% RE, the precision did not exceed 15% RSD and the LLOQ did not exceed 20%.

#### 2.6.5. Stability

The stability of CuB in the plasma was assessed by analyzing QC samples in rat plasma at three concentration levels, under the following four conditions: (1) long-term stability, QC samples (*n* = 5) were stored at −80 °C for 20 days; (2) short-term stability, QC samples (*n* = 5) were stored at room temperature for 12 h; (3) stability to three freeze–thaw cycles, QC samples (*n* = 5) were detected after three cycles of freezing (−40 °C) and thawing (room temperature); (4) QC samples (*n* = 5) were placed in the autosampler for 24 h at 4 °C after being prepared. After the four tests, the measured stability was within the allowable range of accuracy (±15%) and precision (15% RSD).

### 2.7. Pharmacokinetics study

Twelve male Wistar rats weighing 240–280 g were subjected to two different treatments. The first group received an oral administration of 8 mg kg^−1^ CuB suspended in a 0.5% CMC-Na aqueous solution. The second group was intravenously injected with CuB at a dose of 1.3 mg kg^−1^ dissolved in *N*,*N*-dimethylacetamide–PEG 400–water (2 : 4 : 1, v/v/v). After 0.083, 0.25, 0.5, 0.75, 1, 2, 3, 4, 5, 6, 8, 10, 12, and 24 hours post injection, a 0.3 mL blood sample was collected through the posterior orbital venous sinus and transferred to heparinized tubes. All of the samples were immediately centrifuged at 15 000 rpm for 10 min to separate the plasma, and then stored at −80 °C until analysis. With the help of DAS (Drug and Statistics) software (version 3.2.8, Chinese Mathematical Pharmacology Specialized Committee, Shanghai, China), a non-compartment model was used to calculate pharmacokinetics parameters. Values were expressed as the mean ± the standard error of the mean (SD).

### 2.8. Absolute bioavailability

The absolute bioavailability (*F*) was calculated using the formula:^21^*F* (%) = (AUC_i.g._) × (dose_i.v._)/(AUC_i.v._) × (dose_i.g_) × 100%AUC_i.g._ and AUC_i.v._ represented the areas under the concentration–time curves of the analytes after CuB was administrated orally and intravenously to the rats, respectively. Dose_i.g._ and dose_i.v._ represented the dose of the analytes administered orally and intravenously to the rats.

## 3. Results and discussion

### 3.1. Selectivity

**Fig. 2:**
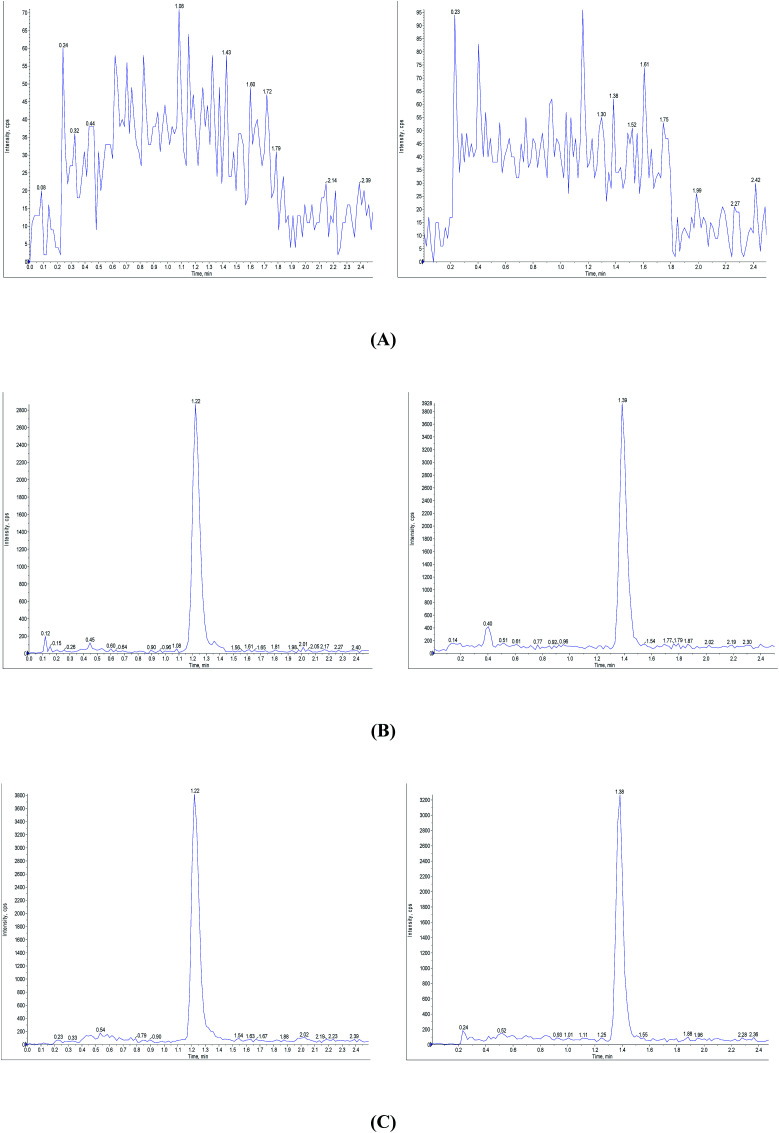
Representative chromatograms of: (A) blank plasma; (B) blank plasma spiked with CuB (2 ng mL^−1^ for CuB) and estrone (IS); and (C) a representative plasma sample 0.5 h after drug administration. Left and right are the MRM scans for CuB and the IS, respectively.

The selectivity was examined by analyzing blank plasma samples from six different rats. Under the LC-MS/MS conditions, no significant interference peaks from endogenous substances were observed in the retention regions of CuB and the IS. This data demonstrated the good selectivity of the LC-MS/MS method. Typical chromatograms are shown in Fig. 2.

### 3.2. Linearity and LLOQ

The linear equation of the calibration curve, linear range and LLOQ of the analyte were measured by the LC-MS/MS method. The assay showed good linearity in the range of 0.05–1000 ng mL^−1^ of CuB. The equation for the calibration curve was: *y* = 16 234.5*x* + 3365.9, *r* = 0.9998. This value proved that the analyte had good linearity. *x* represented the plasma concentration while *y* represented the ratio of the CuB peak area to that of the IS. The LLOQ of CuB was 0.05 ng mL^−1^. The accuracy and precision of the LLOQ were all in accordance with requirements (less than 20%).

### 3.3. Matrix effects, recovery, accuracy and precision

Under the three QC concentration levels, the matrix effects of CuB in rat plasma ranged from 94.19% to 99.42%, while those of the IS were 100.83%. The RSD in these analyzed samples was <7.0%, which was in accordance with regulations. Therefore, no significant matrix effect was observed for the analytes. The extraction recoveries from plasma were 85.34–90.53%. The mean recovery of the IS from rat plasma was 96.38% using this method. The observed data suggested that the sample treatment was suitable for the method. The data for the matrix effects and recoveries are shown in Table 1.

**Table 1:** Matrix effects and extraction recoveries of CuB and IS in rat plasma (*n* = 6)

Ingredient	Conc. (ng mL^−1^)	Matrix effect		Extraction recovery	
		Mean (%)	RSD (%)	Mean (%)	RSD (%)
CuB	0.1	96.35 ± 3.15	3.27	89.44 ± 5.19	6.22
	25	99.42 ± 3.23	3.24	90.53 ± 3.65	4.03
	750	94.19 ± 4.14	4.39	85.34 ± 3.55	4.16
IS	848	100.83 ± 6.16	5.44	96.38 ± 5.82	3.91

Accuracy and precision values were obtained by measuring the QC samples in six replicates at four concentration levels. The results from three consecutive days are shown in Table 2. The absolute values of the RE for the intra- and inter-day accuracy were <10%. The intra- and inter-day precision was below 15% with a maximum RSD of 10.46%. All of the results were within a satisfactory range, which showed that the method had good operability and reproducibility. Therefore, the method was suitable for the determination of constituents in plasma samples.

**Table 2:** Accuracy and precision values for the determination of CuB (*n* = 6)

Conc. (ng mL^−1^)	Accuracy (RE%)		Precision (RSD%)	
	Intra-day	Inter-day	Intra-day	Inter-day
0.05 (LLOQ)	5.32	6.09	6.24	10.46
0.1	3.17	−2.52	7.89	8.64
25	1.77	3.93	2.90	6.25
750	3.53	−4.03	1.77	1.68

### 3.4. Stability

Under the following storage conditions, the content of CuB in the three concentrations of rat plasma was stable. The storage conditions were as follows: preservation at −80 °C for 20 days, storage at room temperature for 12 h, 3 freeze–thaw cycles or 4 °C in the autosampler stored for 24 h. The outcomes of the stability tests are summarized in Table 3.

**Table 3:** Stability for the determination of the three concentrations in rat plasma (*n* = 5)

Storage conditions	Accuracy/precision (mean ± SD/RSD%)		
	0.1 (low QC)	25 (mid QC)	750 (high QC)
Long-term stability at −80 °C for 20 days	0.1 ± 0.008/5.27	24.3 ± 0.62/4.85	754 ± 2.6/3.19
Short-term stability at room temperature for 12 h	0.1 ± 0.011/6.19	25.7 ± 0.38/5.79	751 ± 5.7/3.53
Freeze–thaw stability, three cycles	0.09 ± 0.003/4.78	24.8 ± 0.52/5.02	747 ± 4.9/3.91
Autosampler stability at 4 °C for 24 h	0.11 ± 0.003/5.71	25.3 ± 0.72/3.71	755 ± 2.3/2.96

### 3.5. Optimization of UPLC conditions

The pretreatment of plasma samples is one of the factors that influences the determination of analytes in plasma. Due to the small sample volume, low concentration and highly interfering components of plasma samples, it is necessary to establish appropriate treatment methods to ensure the accuracy, scientificity and effectiveness of analyte determination. In the previous study, liquid–liquid extraction was used to purify components and improve sensitivity. The choice of extraction solvents is a critical step in the pretreatment of plasma samples. Extraction solvents include ethyl ether, dichloromethane, ethyl acetate and chloroform, all of which had different effects on the extraction and recovery rates of CuB. The extraction rate of ethyl acetate was significantly higher than that of the other organic solvents, without interference from endogenous substances and good reproducibility. Therefore, the liquid–liquid extraction method with ethyl acetate was finally selected for the sample pretreatment.

In order to improve the peak shape, sensitivity, and selectivity and to shorten run times, chromatographic conditions should be optimized. The mobile phases consisted of methanol–water, acetonitrile–water, methanol–0.1% formic acid, acetonitrile–0.1% formic acid and other elution systems. Among these, acetonitrile–0.1% formic acid was chosen as the best liquid phase due to producing a higher signal response and having a better peak shape than the other combinations. Finally, a good chromatographic separation was obtained under the elution conditions with acetonitrile–0.1% formic acid at a flow rate of 0.3 mL min^−1^. The method has a short running time of 2.5 min, which allows for more samples to be determined.

### 3.6. Pharmacokinetics study

**Fig. 3:**
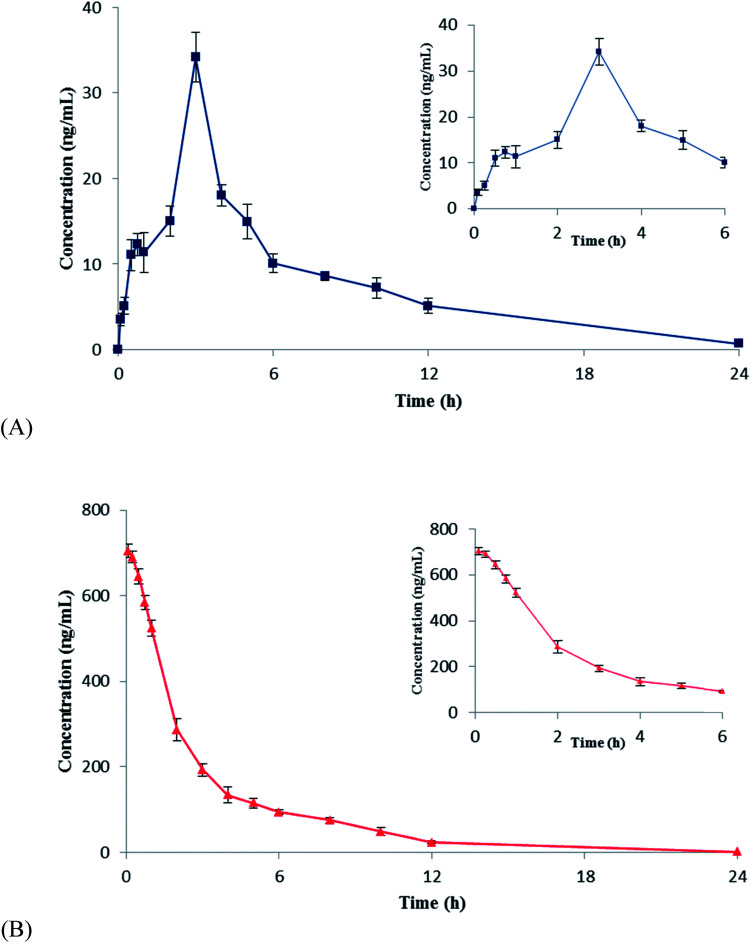
Mean plasma concentration–time curves of CuB in rats after oral administration (A) and intravenous administration (B) of CuB. Each point represents the mean ± SD (*n* = 6).

The validated UPLC-MS/MS method was successfully applied for the quantitative determination of CuB after single-dose administration in rats. All of the measured concentrations were higher than the LLOQ in the plasma. Mean ± SD plasma concentration–time curves of CuB after administration are shown in Fig. 3. The main pharmacokinetics parameters of CuB were identified and are listed in Table 4. Double peaks were observed in the curves of the plasma concentration–time profiles of CuB after oral administration of CuB. After oral administration of CuB in rats, the AUC_0–*t*_ of CuB in rat plasma was 183.28 ± 10.24 ng h^−1^ L^−1^. *T*_max_, *C*_max_, and *t*_1/2_ were 3 h, 34.16 ± 2.91 ng mL^−1^, and 4.129 ± 0.54 h, respectively. Yet, the AUC_0–*t*_ of the plasma parameters of the CuB injection was 2181.25 ± 42.49 ng h^−1^ L^−1^. *T*_max_, *C*_max_, and *t*_1/2_ were 0.117 ± 0.075 h, 706.55 ± 14.58 ng mL^−1^ and 2.77 ± 0.28 h, respectively (Table 4). Compared with the previous methods, the approach adopted in the present article has higher experimental sensitivity and simplicity. The LLOQ was only 0.05 ng mL^−1^. In the previous study, Zhao *et al.* found that the elimination half-life (*t*_1/2_) of CuB was 2.50 ± 0.58 h,^16^ and Wang *et al.* found that CuB in cucurbitacin tablets *t*_1/2_ was 3.19 ± 0.54 h,^17^ while the *t*_1/2_ of CuB was 4.129 ± 0.54 h in this paper. Double peaks were observed in the curves of mean plasma concentration–time profiles of CuB after oral administration of CuB. This is similar to the Zhao *et al.* and Wang *et al.* studies after oral administration of CuB or cucurbitacin tablets. The reason for the double peaks phenomenon of CuB may be attributed to distribution reabsorption and enterohepatic circulation. It is known that the oral CuB has a low bioavailability problem; in the present study the absolute bioavailability of oral administration of CuB in rats was 1.37%. The reasons leading to low absolute bioavailability are drug solubility, membrane permeability, gastrointestinal digestion and absorption. Improvement of the bioavailability is of utmost importance, and there are many ways to improve the bioavailability of oral drugs, including increasing the water solubility of drugs, inhibiting the intestinal metabolism and inhibiting the transport of intestinal mucosal carriers (such as P – protease inhibitors).

**Table 4:** Pharmacokinetics parameters of CuB after administration to rats (*n* = 6)

Parameters	Unit	i.g. (8 mg kg^−1^)	i.v. (1.3 mg kg^−1^)
AUC_0–*t*_	ng L^−1^ h^−1^	183.28 ± 10.24	2181.25 ± 42.49
AUC_0–∞_	ng L^−1^ h^−1^	187.41 ± 10.41	2187.11 ± 42.07
MRT_0–*t*_	h	6.49 ± 0.18	3.65 ± 0.068
MRT_0–∞_	h	7.02 ± 0.29	3.72 ± 0.09
*t*_1/2z_	h	4.129 ± 0.54	2.77 ± 0.28
*T*_max_	h	3	0.117 ± 0.075
*V*_z_/*F*	L kg^−1^	2.55 × 10^8^ ± 3.62 × 10^7^	2.38 × 10^6^ ± 2.47 × 10^5^
CL_z_/*F*	L h^−1^ kg^−1^	4.28 × 10^7^ ± 2.47 × 10^6^	5.95 × 10^5^ ± 1.15 × 10^4^
*C*_max_	ng L^−1^	34.16 ± 2.91	706.55 ± 14.58

In a future study, we will prepare solid dispersions to improve solubility, using poloxamer as a polymeric carrier. The solid dispersion will be evaluated *in vitro* and *in vivo* using rats. The comprehensive data in our current study indicate that the method is applicable to the pharmacokinetics study of CuB in rats.

## 4. Conclusions

This study established and validated a highly selective and sensitive UPLC-MS/MS method which can be used to detect lower levels of CuB in rat plasma; the standard curve is over a concentration range of 0.05–1000 ng mL^−1^. The conditions for the mobile phase and sample extraction solvent were optimized. The presented method has been validated on plasma samples in experimental animals. Nevertheless, the absolute bioavailability of orally administered CuB in rats was only 1.37%. Therefore, the absolute bioavailability of orally administered CuB is an urgent and challenging issue for most researchers. In addition, although the present study provided the data and theoretical basis for the application of CuB, further studies on bioavailability are necessary.

## Ethical statement

This study was performed in strict accordance with the NIH guidelines for the care and use of laboratory animals (NIH Publication No. 85-23 Rev. 1985) and was approved by the Animal Experimental Ethics Committee of Chengde Medical College (No. CDMULAC-2017-003, Chengde, China).

## Conflicts of interest

The authors declare that they have no conflicts of interest.

## Abbreviations

CuB: Cucurbitacin B
IS: Internal standard
UPLC-MS/MS: Ultra-performance liquid chromatography-mass spectrometry
LLOQ: Lower limit of quantification

## Supplementary Material
